# *iMAX* FRET (Information Maximized FRET) for Multipoint Single-Molecule Structural Analysis

**DOI:** 10.1021/acs.nanolett.4c00447

**Published:** 2024-07-08

**Authors:** Bhagyashree S. Joshi, Carlos de Lannoy, Mark R. Howarth, Sung Hyun Kim, Chirlmin Joo

**Affiliations:** † Kavli Institute of Nanoscience, Department of Bionanoscience, Delft University of Technology, Delft 2629HZ, The Netherlands; ‡ Department of Biochemistry, University of Oxford, South Parks Road, Oxford OX1 3QU, U.K.; ∥ Department of Physics, Ewha Womans University, Seoul 03760, Republic of Korea; ⊥ New and Renewable Energy Research Center, Ewha Womans University, Seoul 03760, Republic of Korea

**Keywords:** single-molecule FRET, single-molecule structural analysis, single-molecule conformational analysis, computational structure prediction, programmable DNA binding

## Abstract

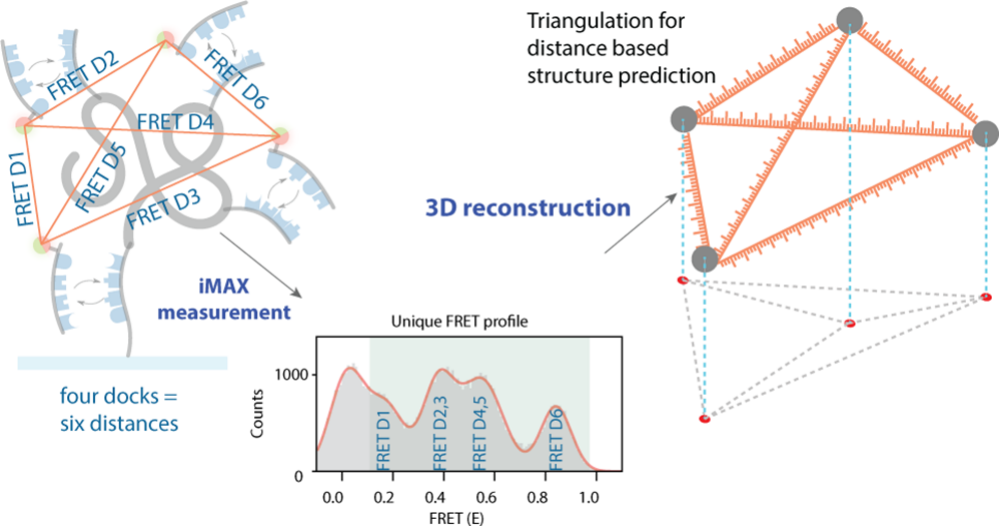

Understanding the structure of biomolecules is vital for deciphering their roles in biological systems. Single-molecule techniques have emerged as alternatives to conventional ensemble structure analysis methods for uncovering new biology in molecular dynamics and interaction studies, yet only limited structural information could be obtained experimentally. Here, we address this challenge by introducing *iMAX* FRET, a one-pot method that allows *ab initio* 3D profiling of individual molecules using two-color FRET measurements. Through the stochastic exchange of fluorescent weak binders, *iMAX* FRET simultaneously assesses multiple distances on a biomolecule within a few minutes, which can then be used to reconstruct the coordinates of up to four points in each molecule, allowing structure-based inference. We demonstrate the 3D reconstruction of DNA nanostructures, protein quaternary structures, and conformational changes in proteins. With *iMAX* FRET, we provide a powerful approach to advance the understanding of biomolecular structure by expanding conventional FRET analysis to three dimensions.

Three-dimensional structure dictates the functions of biomolecules.^1^ Thus, their analysis is fundamental to understanding their biological functions. Seemingly small perturbations—such as an amino acid substitution, temperature changes, or intramolecular interaction—can lead to profound structural changes, potentially leading to diseased cellular states.^2−6^ Analyzing the structures of individual single molecules and complexes is a prerequisite to understanding all cellular functions. However, traditional analysis techniques such as nuclear magnetic resonance and X-ray crystallography determine only the ensemble-averaged structure^7,8^ and are unable to capture the structure variation of individual molecules that may underpin crucial biological processes. Furthermore, these methods often impose artificial conditions during measurements (such as crystallization)^9−11^ requiring complex methodology.^12,13^ Single-molecule techniques such as single-molecule fluorescence resonance energy transfer (smFRET) and single-particle cryoelectron microscopy have emerged as cutting-edge techniques for interrogating structures. While the complex workflow and reliance on specialized experts of single-particle cryoEM hamper its cross-domain adaptability, smFRET is arguably less complex in execution and more accessible. smFRET can measure distances between fluorescent dye pairs attached to a biomolecule in the 2–10 nm range and has been successfully used for conformational and kinetic analyses of biomolecules.^14,15^ However, due to the signal complexity, only one or two dye-labeled points in a single molecule can be tracked at a time,^16^ precluding a comprehensive understanding of the three-dimensional perspective without prior knowledge of the molecular structure.

In our previous study, we developed FRET X, an extension of conventional smFRET that allows multidistance observations between a single reference position and several monitoring positions within a molecule.^17^ Although this multipoint analysis mitigated some limitations of the conventional smFRET, the necessity of a single reference point provides limited information, sufficient only to obtain structural fingerprint of a single molecule but inadequate for *de novo* structural reconstruction. Meanwhile, *de novo* structural reconstruction from smFRET data has been previously demonstrated by using triangulation of positions in three-dimensional space.^18−20^ However, the authors separately prepared and measured a series of samples, each designed to report a distance of different combinations of the points of interest. Consequently, 3D reconstruction was achievable only by combining data sets from ensembles of single molecules. To date, *de novo 3D reconstruction from* a single individual molecule remains unexplored.

We now present information MAXimized FRET (*iMAX* FRET), a one-pot experimental method that measures all possible mutual distance information between multiple points within a single molecule. Unlike hitherto reported methods that require prior structural knowledge to interpret data, *iMAX* FRET is the first method that enables *ab initio* structural analysis solely from smFRET data. The unique “one-pot measurement scheme” for stochastic multipoint sampling, i.e., no multiple repeated measurement with buffer exchange required, is realized by repurposing the probe exchange scheme, which has been utilized in recent studies to overcome photobleaching of organic dyes for long-term kinetics measurement.^21,22^ Using our newly developed software pipeline, we show that *iMAX* FRET data can determine up to six distances from four positions in 3D space, from which the conformation of a molecule can be reconstructed through geometrical modeling. iMAX FRET provides a comprehensive understanding of structural heterogeneity within a biological sample.

## The Principle of *iMAX* FRET

**Figure 1:**
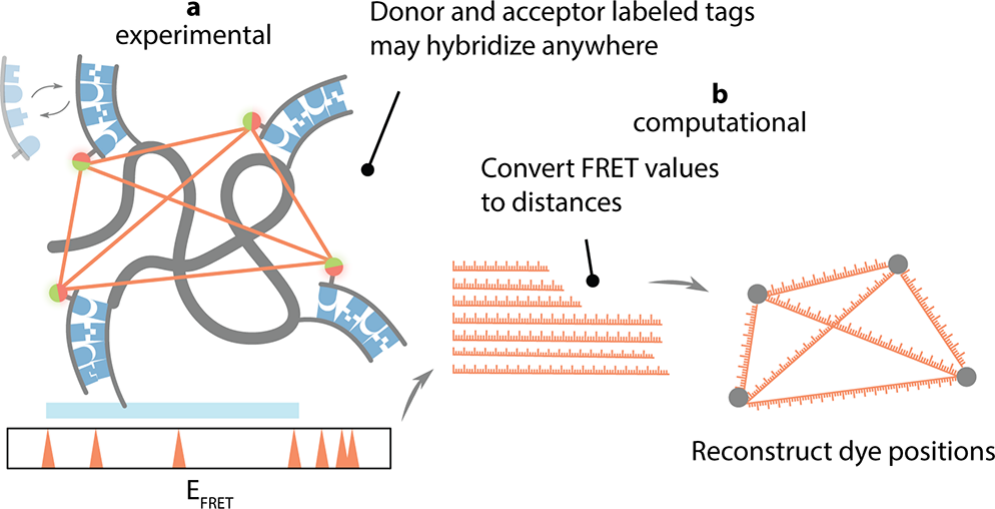
General concept of *iMAX* FRET. **a**, Experimental module. A biomolecule consists of 2–4 coordinates carrying weak binder targets, here DNA docking strands to which cognate imagers can reversibly bind. The imagers are labeled with either donor or acceptor (green as donor and red as acceptor), and they both compete for the binding sites. Each successful FRET event has a particular FRET efficiency (*E*_FRET_) between two coordinates and, over time, all possible FRET efficiencies accumulate to give rise to the FRET histogram. **b**, Computational module. The apparent FRET efficiencies (*E*_FRET_) for single molecules are converted into distances. They are run through geometrical reconstruction to predict the most optimal fit for the structure. This designates the predicted structure calculated based on the apparent FRET efficiencies.

*iMAX* FRET employs weak binders to rapidly assess multiple points in native biomolecules and heteromeric complexes (Figure 1). In this work, we utilized short single-stranded DNA (ssDNA) as weak binders, taking advantage of their programmable binding kinetics. Specific positions of interest within a protein, nucleic acid nanostructure, or multimeric complex were labeled with ssDNA molecules, referred to as docking strands. These docking strands transiently hybridize with complementary DNA oligos in solution, termed imagers, which are labeled with either a donor or acceptor fluorophore (Figure 1a). As these imager binding events occur stochastically and since each docking strand can serve as both the donor and acceptor binding site, all distances between the target positions can eventually be deduced from single-pair FRET events, where only one donor and acceptor imager pair is bound to the target biomolecule. The lengths and concentrations of the imagers are tuned to ensure that only one FRET pair is observed for a significant fraction of the recording time. The collected FRET values are subsequently translated to distances, which are then fit together in a three-dimensional construct (Figure 1b); all possible three-dimensional constructs using these lengths are generated, and the construct that violates the originally measured lengths the least is considered the correct fit. This method allows for per-molecule three-dimensional reconstruction without any prior knowledge of the identity or structure of the molecule; only basic geometry rules are applied.

One advantage of *iMAX* FRET is its relative ease of implementation. A single round of standard two-color FRET measurement is sufficient to obtain all the necessary structural information, whereas other methods for multiple distance observation often require the inclusion and observation of more dye colors, repeated measurements, or multiple sample preparations with different labelings.^16,23−27^

## *iMAX* FRET Can Delineate Single-Stranded DNA Profile

First, we aimed to assess the feasibility of the simultaneous multidistance measurement with the one-pot stochastic probe exchange scheme using ssDNA as a target molecule carrying multiple docking sequences. We prepared four ssDNA targets each of which contains two or three interspaced copies of an otherwise identical docking sequence at different positions, designated A, B, and C (Figure S1a, sequences in Table S1). Simultaneous binding to positions A and B—spaced 12 nt apart—was expected to yield high FRET, B and C were 16 nt apart which should generate an intermediate FRET, and the summed 28 nt distance between positions A and C should result in a low FRET signal (Figure S1a).

A mixture of donor and acceptor imagers of 8 nt was added to the sample chamber containing immobilized targets. The 8 nt imager with binding dwell time τ_binding_ = 1.0 ± 0.1 s was adapted from our previous study.^26^ We added 10-fold excess of acceptor-labeled imagers over donor-labeled imagers to increase the probability of both fluorophores being present simultaneously. All events were collected from time traces of individual molecules (Figure S1b) and we built a histogram of the averaged FRET value/event (Figure S1c). All DNA samples showed the expected FRET efficiencies of 0.73 ± 0.01, 0.52 ± 0.01, and 0.21 ± 0.02 for positions A, B, and C, respectively (Figure S1c). Notably, the DNA sample carrying all three docking sites showed all three peaks, confirming the capability of our stochastic exchange scheme for simultaneous multipoint assessment.

**Figure 2:**
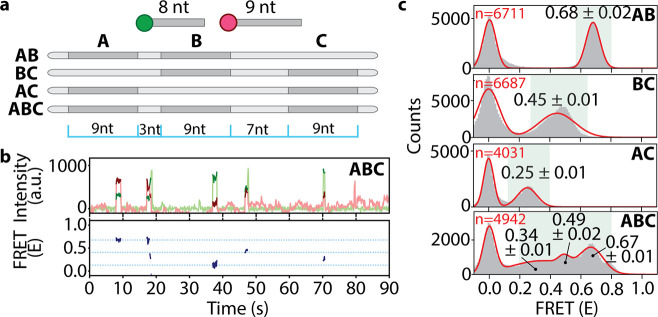
Resolution of three targets in linear DNA using *iMAX* FRET. **a**, Schematic representations of the linear DNA constructs. A, B, and C are the positions of identical docking sequences to which an 8 nt donor- and a 9 nt acceptor-labeled imager can bind. The molar ratio of the donor and acceptor strands were 1:10 (donor: acceptor). AB, BC, and AC are control constructs lacking either one of the three docking sequences, whereas ABC contains all three. The distances between A–B, B–C, and A–C are 12 nt, 16 nt, and 28 nt, respectively. The blue scale explains the distances between A, B, and C. **b**, Representative single-molecule intensity vs time trace (top panel) for the ABC construct (green for donor and red for acceptor intensities). Note that there are three different intensity peaks for the red i.e., acceptor intensity showing FRET events corresponding to successful donor–acceptor imager pair binding to A–C, B–C, and A–C docking sequences. The bottom panel shows the marked FRET efficiencies in blue. The highest blue line corresponds to A–B FRET, the middle line to B–C FRET while the lowest designates the A–C FRET event. **c**, Single-pair FRET event histograms from all molecules in a single field of view (gray bars). The mean FRET ± SEM is given for each peak in the histogram except the peak at 0.0 which corresponds to the donor-only binding events. Red solid lines are multi-Gaussian fit to the histograms. The FRET efficiency of each peak represents the distance between the designated docking sequences. Note the three peaks in the ABC construct corresponding to the three distances for A–C (0.34 ± 0.01), B–C (0.49 ± 0.01), and A–B (0.67 ± 0.01).

We noted that the majority of binding events showed FRET efficiency of 0.0 (star, peak area of ∼69%), indicating that donor-only binding events were still dominant (Figure S1c). Thus, the acquisition of sufficient FRET events, i.e., simultaneous binding of a donor and an acceptor imager, required precise adjustment of the binding kinetics of imagers; event duration controlled by imager lengths, and event frequency controlled by imager concentrations. Using Monte Carlo simulations of our experiment (Supplementary Methods) across various concentrations and binding dwell times, we inferred that employing a 10-fold excess of acceptor combined with longer acceptor binding times produced the optimal number of single FRET-pair events (Figure S1d and e). Indeed, utilizing longer 9 nt acceptor imager with 8 nt donor imager in the experiment led to a substantial increase in viable FRET events (Figure 2a–c, Figure S1f–g). This demonstrated that careful rational design of imager lengths, and hence dwell times, is pivotal in resolving multiple targets in *iMAX* FRET.

## *iMAX* FRET Can Resolve DNA Nanostructures

**Figure 3:**
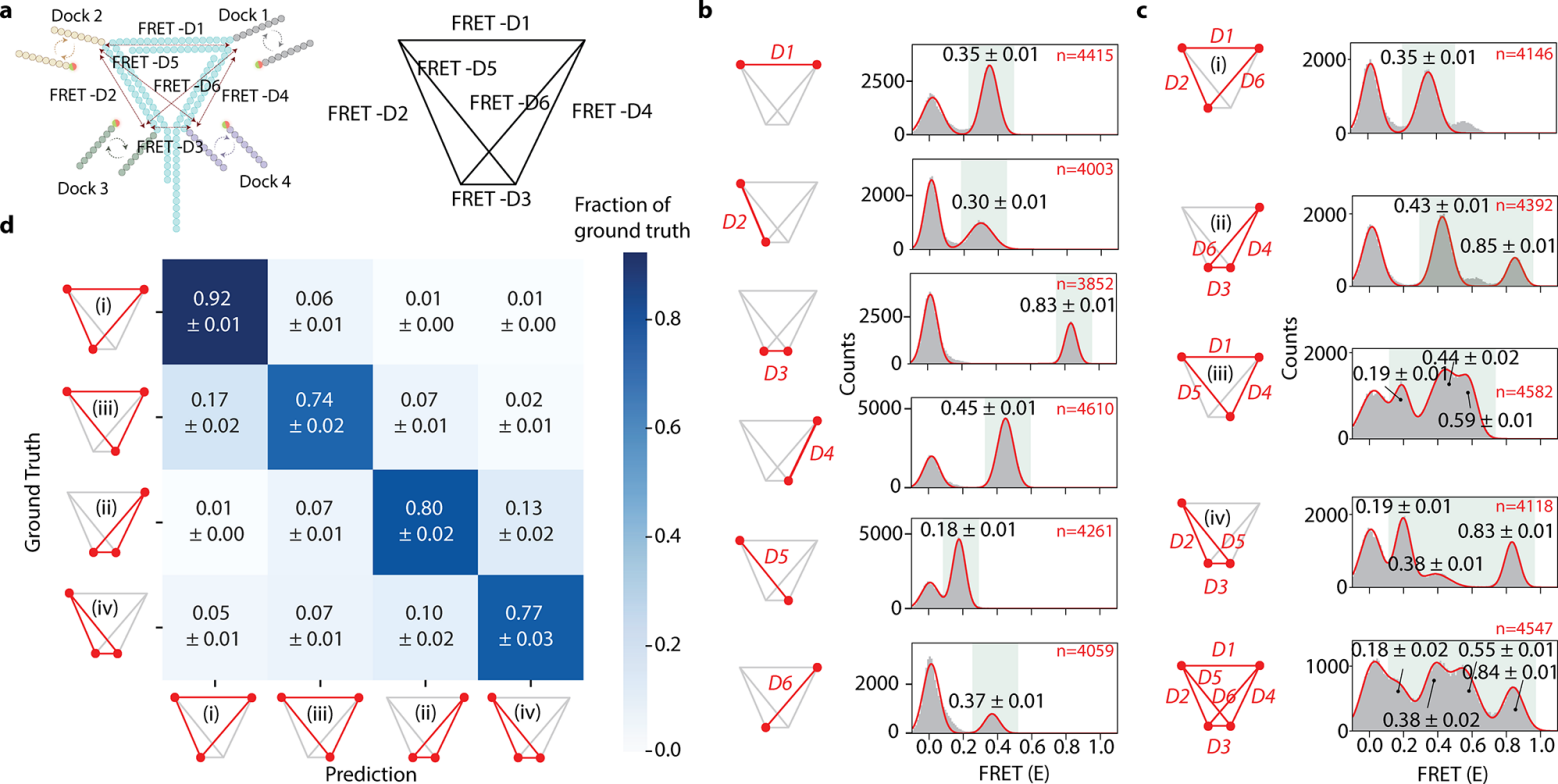
*iMAX* FRET provides structural analysis of a complex DNA nanostructure. **a**, Schematic representation of DNA nanostructure containing 4 overhangs of different DNA sequences which act as docking segments (Docks) for the imagers. As cognate imagers labeled with a donor or acceptor dye bind transiently to the Docks, FRET events occur in proportion to the distances between the Docks. Fifteen bp DNA length separates each pair of Docks 1–2 and 2–3, whereas the 13 bp segment separates the Docks 1–4 giving rise to FRET distances of FRET-D1, D2, and D4, respectively. As a result, Docks 3 and 4 are situated very close to each other giving rise to FRET-D3. The Docks 2–4 and 1–3 also make a pair culminating in FRET-D5 and -D6, respectively. The right panel is the line representation of all the distances generated from the DNA nanostructure and will be used henceforth as a model figure. **b**, *iMAX* FRET histograms of each FRET distance D1 to D6, separately. The red lines signify the FRET distance, red dots represent the Docks. Note that D1, D2, D4, and D6 are similar while FRET D2 and D3 mark the extremes in either direction. The shorter length of DNA (FRET D4, 13 bp) is reflected in slightly higher FRET efficiency (0.45 ± 0.01) as opposed to 2 bp longer FRET D1 and D2 (0.35 ± 0.01 and 0.30 ± 0.01), respectively. This hints at the distorted nanostructure due to differential side lengths. **c**, *iMAX* FRET histograms for combinations of all 3 spatial points forming triangles (i–iv). The red lines signify the FRET distances, red dots represent the Docks. Note that triangle (i) has one mid-FRET degenerate peak due to the three overlapping distances of D1, D2, and D6. Triangle (ii) has one mid-FRET degenerate peak from D4 and D6, and a high-FRET peak arising from D3. The bottom structure contains four peaks with 2 degenerate peaks and 2 single peaks as a result of all the FRET distances D1–D6. **d**, Confusion matrix showing classification accuracy and error modes of a tree-based machine learning classifier trained to identify the four triangles (i–iv) on a single molecule level and tested on held-out molecules. Each row denotes which fraction of total molecules for a given ground truth class are ascribed to which class, where the diagonal denotes correct classifications (i.e., the per-class accuracies).

To demonstrate *iMAX* FRET’s capability of *ab initio* three-dimensional structure determination, we analyzed a quadrangular DNA nanostructure outfitted with a docking strand at each corner (Figure 3a, left). This nanostructure featuring six distinct distances, referred to as D1 to D6 (Figure 3a, right), could be probed with four identical docking strands in *iMAX* FRET in a one-pot reaction. In this demonstration, however, we prepared each docking strand with a unique sequence for control purposes.

First, we probed each distance individually by adding two different imager strands, resulting in a single FRET peak (Figure 3b). We found that D3 and D5 were well-discernible from the other distances (FRET efficiency mean ± standard deviation of 0.83 ± 0.01 and 0.18 ± 0.01, respectively). D1, D2, D4, and D6 generated highly similar FRET values (0.35 ± 0.01 and 0.30 ± 0.01, 0.45 ± 0.01 and 0.37 ± 0.01, respectively). We increased the complexity by adding 3 imager strands for simultaneous analysis of three distances. Indeed, for each of the four possible triangles in this quadrangle, we could identify the expected number of FRET peaks (Figure 3c, panels i–iv). Triangle i (constructed from D1, D2, and D6) showed one major peak, whereas triangle ii (D3, D4, and D6) displayed two overlapping peaks, as expected based on single-distance analysis results. Similarly, triangles iii and iv showed three peaks for (D1, D4, D6), and (D2, D3, D5), respectively.

Next, we probed all six distances simultaneously by adding four different imagers together (Figure 3c, bottom plot) and observed four peaks. The highest at *E* = 0.84 and the lowest at *E* = 0.18 represented D3 and D5 respectively. However, the other two peaks were not straightforward to assign due to the overlapping FRET values of the other four distances D1, 2, 4, and 6. Nevertheless, the broad peak at 0.38 could be assigned as a degenerate peak of D1, D2, and D6, while the peak at *E* = 0.55 likely arose from D4.

Having acquired the distances, the reconstruction of triangle coordinates in Figure 3c is trivial as only one dissimilar triangle (i.e., ignoring rotation, translation, and reflection) can be constructed given the lengths of all three edges. Aligning and averaging triangle coordinates of all single molecules (Figure S2a), produced the shapes of the four triangle types (Figure S2b). To demonstrate that triangles reconstructed for single molecules contain sufficient information to allow recognition, we encoded the coordinates in a rotation-, translation-, and reflection-invariant embedding^28^ and trained a tree-based machine learning algorithm to recognize each type. On held-out molecules, this classifier attained an accuracy of 74%, confirming that spatial reconstruction contains discriminative information (Figure 3d). Most errors were made between triangles that were expected to show more similarity due to the nanostructure’s assumed symmetry (i+iii, ii+iv respectively). Even so, the classifier still correctly assigned classes to most molecules, indicating that the nanostructure is not truly symmetric.

Finally, we reconstructed the complete 3D quadrangle by using the FRET values obtained from the six-distances measurement in Figure 3c, bottom plot. In theory, 30 dissimilar quadrangles can be constructed given the lengths of all six edges. However, not all quadrangles can necessarily be built without violating the given lengths. An analysis pipeline was written (Supplementary Methods) that builds all possible dissimilar quadrangles and chooses the one for which the edge lengths are required to change the least to fit. We found that a 3D quadrangle could be constructed satisfying all distances without violating the FRET-derived lengths (Figure S3).

Similar to the triangle reconstructions, this 3D reconstruction indicated that the nanostructure has an asymmetric conformation, rather than a planar symmetric conformation. We surmised that this reflected, in part, the slight out-of-plane attachment positions of the docking strands due to the helical structure of the double-stranded DNA, as well as the flexible carbon linker between the dyes and the DNA. To investigate whether these factors could truly contribute to the observed deviations, we prepared three quadrangles with one of the docking strands positioned at varying positions along the long edge of the structure, and reconstructed triangle shapes for each (Figure S4a–h). The helical nature of the dsDNA should be evident from the respective shapes and sizes of these triangles, as the variably positioned dye moves in and out of the quadrangle plane depending on its distance from the apex. We thus fitted the obtained triangles into the helical structure of dsDNA and calibrated the FRET radius and linker length (Supplementary methods). Satisfactorily, the reconstructed triangles fit the expected dye positions around the DNA helix shape with subangstrom accuracy (Figure S4g, Table S2), indicating that these factors were indeed contributing to the measured quadrangle shape.

In summary, *iMAX* FRET could successfully demonstrate the structural analysis of up to 4 points in a complex DNA nanostructure, and we could predict and retrieve these structural identities with high accuracy based on FRET fingerprints and computational modeling. This demonstrates that we could expand the signal space to 6 peaks (considering degenerate peaks) in a one-pot reaction requiring less than 2 min without using solution exchanges.^26^ While the degeneracy of the FRET values may complicate data interpretation, it is worth noting that the number of observed distances provides a clue on the number of degenerate peaks—if the number of detected distances is not a triangular number (1, 3, 6, etc.), this suggests the requirement for the addition of degenerate peaks until the next triangular number. Systematically trying to add multiples of each distance will result in one or more best structures.

## *iMAX* FRET Locates the Biotin Pockets in Tetravalent and Divalent Streptavidin Structures

As *iMAX* FRET is well-suited to determine the relative position of three or more points in space, we set out to study multimeric structures, which are difficult to analyze with traditional FRET due to the inability to control labeling with donor and acceptor fluorophores of subunits within a multimeric protein.^29^ Structural analysis of multimeric proteins by other techniques, including mass spectrometry, often requires complex stabilization using chemical linkers or cross-linking.^30−33^ In contrast, *iMAX* FRET can be applied to native complexes. Moreover, ligand-binding multimers present a unique possibility for *iMAX* FRET. For example, we can use docking strand-conjugated ligands to probe the positions of their binding pockets. We chose streptavidin as our model protein, as it contains four pockets for biotin. This also allowed us to indirectly immobilize streptavidin to a surface, by occupying one of its pockets with an immobilized biotinylated docking strand (Figure S5a). The other pockets were occupied by docking strands added to the solution.

**Figure 4:**
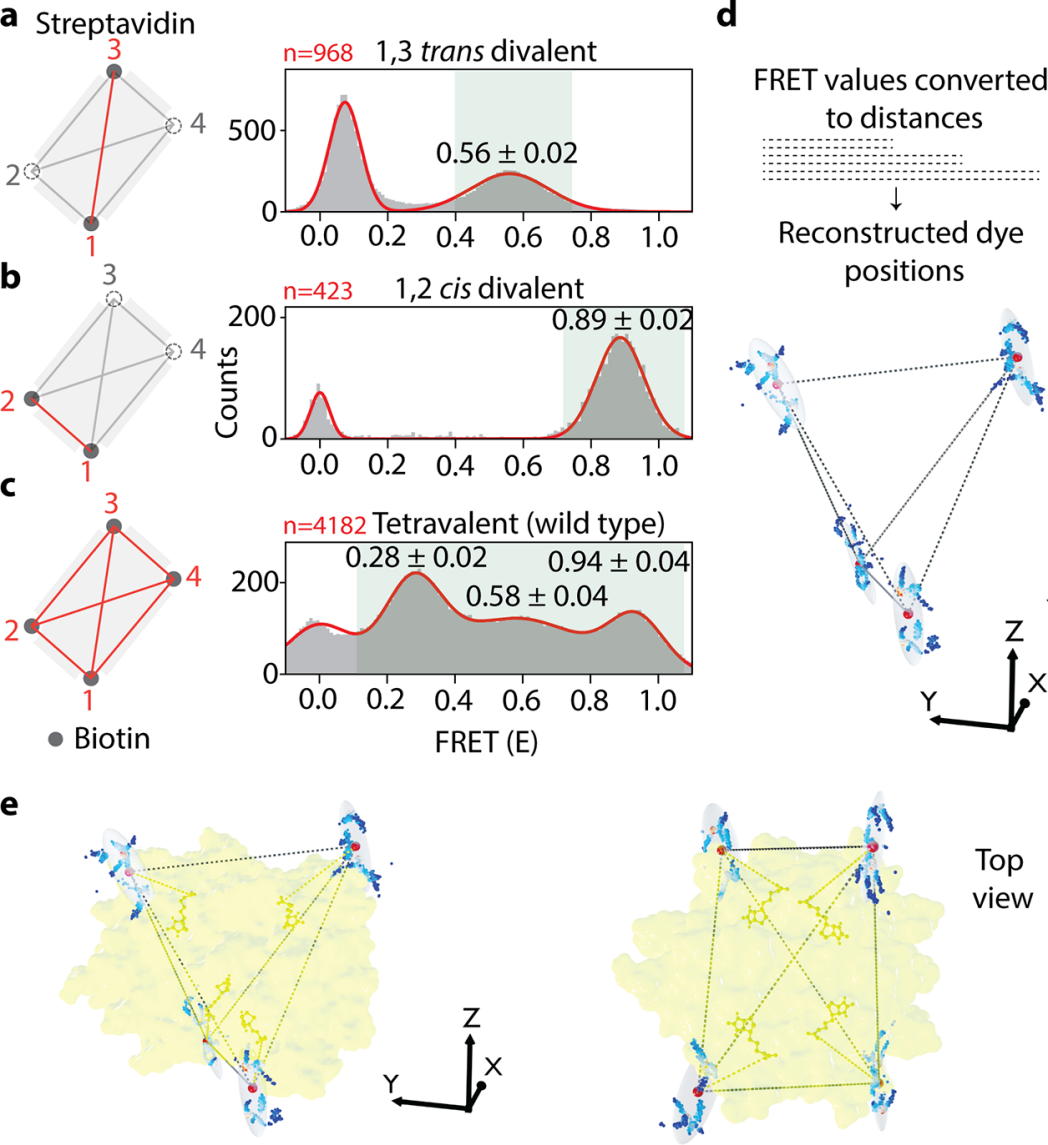
*iMAX* FRET-based structural analysis of streptavidin complexes. **a**, The mutant *1,3 trans* divalent streptavidin (PDB ID: 4BX6) can bind biotin (gray dots) to only two binding pockets, whereas the other two are mutated to abrogate the biotin-binding (dashed circles). Upon binding of an imager, the dye is facing toward the binding pocket. The distance between the bound biotins (red line) shows the FRET efficiency of 0.56 ± 0.02 in the histogram. **b**, The mutant 1,2 *cis* divalent streptavidin (PDB ID: 4BX5) can bind biotin as shown. The distance between the bound biotins (red line) shows a high FRET efficiency of 0.89 ± 0.02. **c**, The wild-type tetravalent with four active biotin-binding pockets. Hence, it can give rise to six distance possibilities [*n*(*n* – 1)/2]. However, streptavidin is a symmetrical molecule, hence shows three degenerate peaks, each peak corresponding to two overlapping peaks. **d**, The FRET values are converted to six distances, and a structure is reconstructed for four biotin-binding pockets. Average positions for 1000 bootstrap iterations over all molecules are shown as dots (colored by density), the mean position is shown as a large red sphere and ovals report one standard error intervals (on average, 2.75 Å). **e**, The reconstructed structure is fitted into the reported (PDB ID: 2IZF) crystallographic streptavidin:biotin structure (yellow). Note that all four biotins can be fitted into the biotin-binding pockets with high accuracy.

Streptavidin is a tetramer organized in a tetrahedral (D2) symmetry with four biotin-binding pockets (Figure S5b). To derive single distances from four pockets, we measured two divalent streptavidin mutants—1,3 *trans* and 1,2 *cis* having only two active biotin binding pockets^34^ (Figure 4a and b). As expected, a high FRET peak (0.89 ± 0.04) was observed for 1,2 *cis* and a mid-FRET peak (0.56 ± 0.02) for 1,3 *trans* (Figure 4a and b). Changing the dye positions from one end to another of the imagers proportionately reflected the changes in the FRET values, showing the ability of *iMAX* FRET to pinpoint the biotin-binding pockets accurately (Figure S5c and d). Subsequent *iMAX* FRET analysis on the wild-type streptavidin with four active binding pockets showed three different FRET efficiencies of 0.28 ± 0.02, 0.58 ± 0.04, and 0.94 ± 0.04 seen for pockets 1, 2, and 3, respectively (Figure 4c). Although, six distances were expected, the symmetric tetramer structure of streptavidin could exhibit only three peaks due to degeneracy. Nevertheless, by using these FRET values, we were able to reconstruct the relative positions of the binding pockets (Figure 4d). The reconstructed 3D spatial coordinates fit the known streptavidin structure gratifyingly well, accounting for a realistic average linker length of 1.8 nm, and showed limited variability over 1000 bootstrap iterations (SD of 2.75 Å averaged over all positions, Figure 4e). This confirms that *iMAX* FRET is capable of extracting three-dimensional features from multimeric proteins without the aid of complementary methods or additional information.

## *iMAX* FRET Can Be Potentially Used to Analyze Protein Conformational Changes

Next, we explore the compatibility of *iMAX* FRET with studying conformational changes of proteins without disturbing their activity. Many proteins undergo profound conformational changes upon binding to a ligand. A well-known example is substrate binding domain (SBD)^35^ which captures extracellular substrates and delivers them to transporters. We focused on Gln *P*Q from *Lactococcus lactis*, involved in amino acid sensing and import of asparagine and glutamine.^36,37^ Here, we attempted to detect the open-to-closed conformational switch after ligand binding to the SBD2 protein.^38,39^

**Figure 5:**
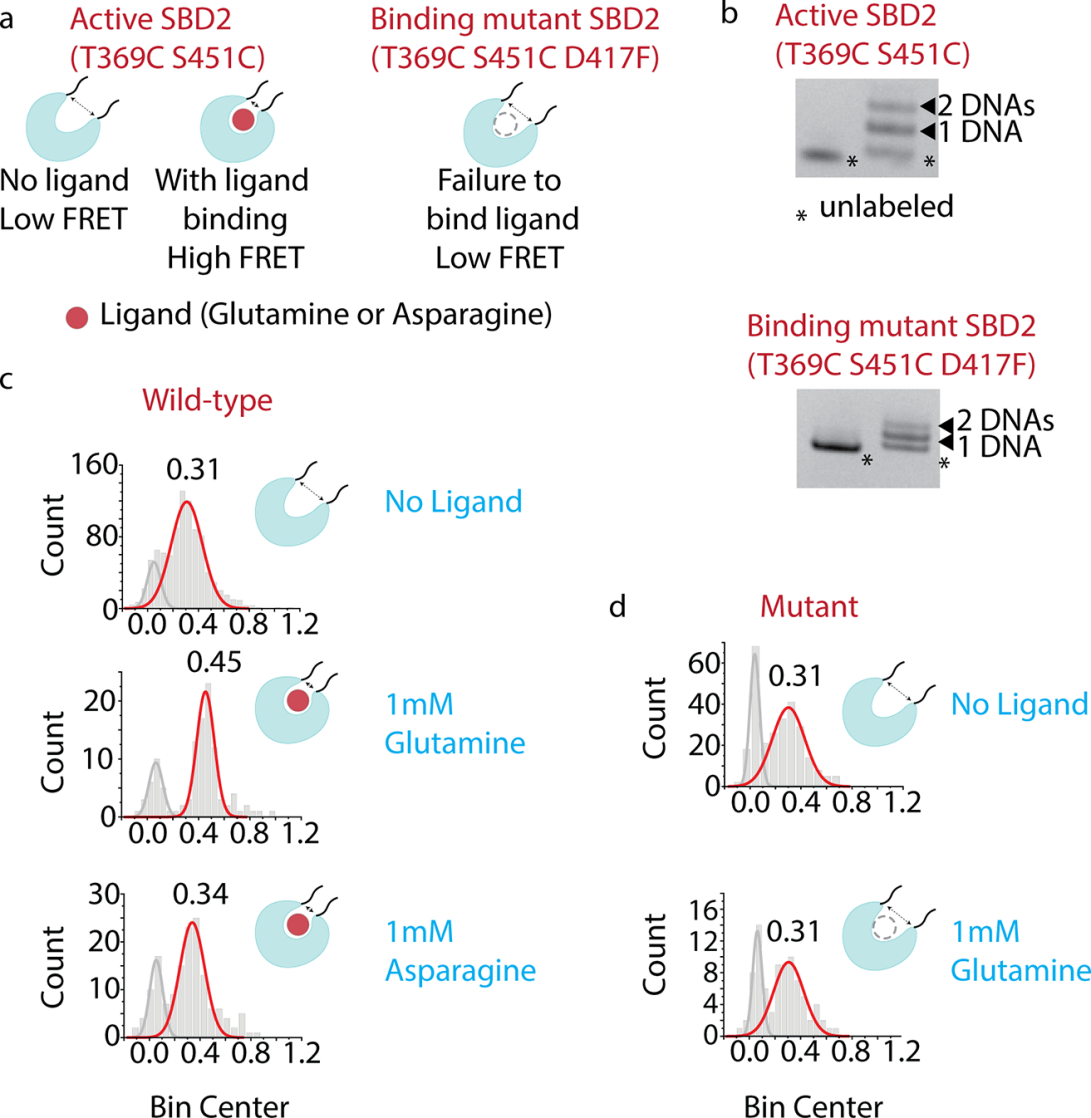
Structural analysis of conformational changes in SBD2-ligand complexes. **a**, 2 mutant SBD2 proteins—active (T369C S451C) and null (T369C S451C D417F). The cysteines are strategically added for DNA labeling. When a cognate ligand is bound, the conformation change results in higher FRET, whereas the null mutant retains the low-FRET value due to a lack of ligand binding. **b**, The SBD2 proteins are labeled with DNA using click chemistry. The ladder pattern suggests the weight shift due to the addition of one or both DNAs attached to the protein. **c**, The SBD2 protein changes its 0.31 FRET value (no ligand) to 0.45 upon its preferred glutamine ligand binding. When Asparagine is added, it stabilizes at 0.34 FRET. **d**, The mutant SBD2, due to the inability of ligand binding, remains at 0.31 FRET after the application of glutamine.

A wild-type protein that can bind glutamine and asparagine and a null mutant that does not bind any ligand as control were prepared^39^ (Figure 5a). For DNA labeling, two cysteines were inserted into both proteins at strategic positions with no known adverse effects,^39^ each located at one of the two lobes in SBD2 (Figure 5b). The distance between these two positions undergoes a significant change after a ligand binding according to the crystal structures.^38^ Indeed, observed FRET increases from 0.31 to 0.45 and from 0.31 to 0.34 upon binding of Glutamine and Asparagine, respectively (Figure 5c). The smaller FRET shift with Asparagine reflected the fact that SBD2 undergoes a higher conformational change when bound to Glutamine compared to Asparagine.^39^ In contrast, we did not observe a FRET shift from the mutant, confirming the FRET shift is indeed induced by ligand binding (Figure 5d). We conclude that *iMAX* FRET with the stochastic DNA probe exchange method can apply to dynamic structural analysis of proteins as a response to stimuli.

In this study, we presented *iMAX* FRET, a pioneering structural analysis tool designed for probing multiple pairwise distances through the utilization of high-resolution smFRET and a weakly interacting probe seamlessly integrating *iMAX* FRET with geometric modeling for structural inference. Our approach facilitates the comprehensive assessment and prediction of molecular architectures leveraging their distinctive FRET signatures with the ultimate sensitivity of a single molecule measurement. This innovative methodology advances the frontier of *ab initio* structural prediction and unlocks new avenues for investigating conformational dynamics heterogeneity within molecular systems. *iMAX* FRET has many advantages over established techniques. First, as we use the stochastic exchange scheme for probing all possible points in a molecule with otherwise identical probes, the imaging time can be cut down considerably compared to other DNA hybridization-based imaging techniques.^26,27,40^ Probe-labeled samples can be prepared within 24 h,^26^ while weak-binder-based measurement can be completed in as little as 2 min. Second, *iMAX* FRET offers simplicity in sample preparation, circumventing the requirements associated with sample preparation required for other methods, such as crystallization. With only picomolar-range quantities needed, this approach facilitates the analysis of precious samples including patient-derived materials. Additionally, *iMAX* FRET allows for the interrogation of multiple distances in a nano object, including complex DNA nanostructures, proteins, and heteromeric complexes. It paves the way for studying the static and dynamic structural analysis of challenging multimeric proteins such as transcription factors and transmembrane proteins. Furthermore, *iMAX* FRET has the potential to provide quantitative insights into the species abundance of multimers and their characteristics within complex mixtures of homo and heteromers. Thus, it can replace cumbersome biochemical assays used to delineate the differential populations of homomers and heteromers present in a particular solution. Lastly, with recent developments on the incorporation of constraints into AlphaFold,^41^ the information provided by *iMAX* FRET can be used to attain more accurate de novo structural predictions of any protein. This would be especially useful to resolve conformations of difficult-to-predict dynamic entities such as intrinsically disordered proteins.

Presently, *iMAX* FRET is optimally suited for structural determination within a restricted framework, exemplified by its application to DNA nanostructures and rigid proteins in this study. It is worth noting that DNA conjugation to proteins may induce structural alterations. To avoid this, a rational design strategy for positioning DNA strands on exposed surface of the biomolecules guided by the known structures may be necessary. Further, a functional assay should be performed to confirm that the structure and activity of DNA-conjugated biomolecules are preserved. Moreover, certain challenging scenarios, such as proteins with (1) excessively large or complicated structures, (2) a low or high number of possible labeling points, or (3) considerable degeneracy in the measured distances, may be analyzed over iterative rounds of measurements by utilizing the programmable nature of a probe. Despite these enhancements, *iMAX* FRET would still be limited to molecules where all spatial points under investigation fall within the FRET detectable distance range. Of note is that the range of distances can also be covered by using different FRET pairs, such as Cy2-Cy3 or Cy5-Cy7 pairs, which offers different FRET radii suitable for shorter or longer distances. Further developments should, therefore, include identifying and evaluating widely applicable methods for orthogonal labeling of docking strands or the use of alternative weak binders that do not require conjugation of labels to a protein.

## Data Availability

Data are available upon reasonable request. All codes are documented and freely available at https://github.com/cvdelannoy/iMAX-FRET.

## Supporting Information Available

Materials and methods, additional experimental details, bioinformatic analysis including Monte Carlo simulations, and structural prediction pipelines (PDF)
