# Supplementary material for: iMAX FRET (Information Maximized FRET) for Multipoint Single-Molecule Structural Analysis
Source: Nano Lett. 2024 Jul 8;24(28):8487–94. doi: 10.1021/acs.nanolett.4c00447 (PMC11261617; doi:10.1021/acs.nanolett.4c00447)
Supplement: Supplementary file 1 — nl4c00447_si_001.pdf [file nl4c00447_si_001.pdf]

Supplementary Materials for

iMAX FRET (Information Maximized FRET) for Multipoint Single-Molecule Structural Analysis

Bhagyashree S. Joshi[a], Carlos de Lannoy[a], Mark R. Howarth[b,c], Sung Hyun Kim[a,d,e*], Chirlmin Joo[a,d*]

[a]Kavli Institute of Nanoscience, Department of Bionanoscience, Delft University of Technology, Delft, 2629HZ, The Netherlands
[b]Department of Biochemistry, University of Oxford, South Parks Road, Oxford OX1 3QU, UK
[c]Current address: Department of Pharmacology, University of Cambridge, Tennis Court Road, Cambridge, CB2 1PD, UK
[d]Department of Physics, Ewha Womans University, Seoul 03760, Republic of Korea
[e]New and Renewable Energy Research Center, Ewha Womans University, Seoul 03760, Republic of Korea

* Corresponding authors:
Sung Hyun Kim, ifolium@gmail.com;
Chirlmin Joo, c.joo@tudelft.nl

This PDF file includes:

Materials and methods
Supplementary text including Monte Carlo simulations and structural prediction pipelines
Figures S1 to S5
Supplementary Tables S1 and S2
References

Materials and Methods

Protein expression and purification

Divalent streptavidins were expressed in *Escherichia coli*, refolded from inclusion bodies, and purified by ammonium sulfate precipitation and ion-exchange chromatography, as reported in the original paper[1]. Tetravalent recombinant (wild-type) streptavidin was procured from Thermo Scientific. Plasmids encoding SBD2 (T369C/S451C) and SBD2 (T369C/S451C/D417C)[2] were a generous gift from Prof. Bert Poolman (Department of Biochemistry, University of Groningen, The Netherlands), and the proteins were expressed and purified using the reported protocol [2].

Protein labeling

Cysteine labeling was carried out as reported previously[2] with slight modifications as follows. Cysteine residues of purified proteins (25μM in the total volume of 50μl In PBS) were reduced with 50mM Tris(-2-carboethyl)phosphine (TCEP) at 40-fold molar excess for 30 minutes. Excess TCEP was removed with ZebaTM Spin desalting columns 7kDa MWCO (ThermoFisher) as it may interfere with the Maleimide reaction[3]. The proteins were then labeled with 25-fold molar excess monoreactive maleimide-Dibenzocyclooctyne (DBCO) (Sigma Aldrich) in Phosphate Buffered Saline (PBS) pH 7.4 overnight at room temperature. Excess maleimide-DBCO was removed with Zeba columns and reacted with 10-fold molar excess (ratio 1:10, cysteine to linker) of monoreactive Azidobenzoate-(5') functionalized DNA in PBS pH 7.4 and incubated overnight at room temperature.

Single-molecule Setup

All *iMAX* FRET measurements were performed on a custom-modified prism-type TIRF microscopy setup built around an inverted fluorescence microscope (Nikon, Ti2e)[4]. For illumination of samples immobilized on a quartz slide surface, a 532 nm diode-pumped solid-state laser and 640 nm diode laser (Oxxius, L6Cc) were directed to the surface with an incidence angle below the critical angle via a prism installed above the slide. Fluorescence signals of Cy3 and Cy5 dyes collected by an objective lens (Nikon, CFI Plan Apochromat VC 60X WI) placed below the quarts sample chamber were spectrally divided by a dichroic mirror (Chroma, T635lpxr) after removing scattered laser light by a laser blocking filter (Semrock, NF03-405/488/532/635E-25). The fluorescence signals were further cleared by bandpass filters (Chroma, ET585/65m for Cy3 and ET655LP for Cy5) and imaged on a sCMOS camera (Photometrics, PrimeBSI). The two lasers were operated with a trigger signal generated by the sCMOS camera for ALEX illumination scheme[5]. All the instruments were controlled by using commercial software (NIS elements, Nikon).

Single-molecule flow cell preparation and data acquisition

All single-molecule FRET experiments were performed at room temperature. The flow cells were prepared using our published protocol[6]. Briefly, quartz slides (G. Finkerbeiner Inc) were etched using acidic piranha and passivated with polyethylene glycol (PEG) to minimize any non-specific binding of molecules. mPEG-SVA and PEG-Biotin (Layson Bio) were used for the PEGylation. 50μl of 0.1mg/ml streptavidin (Thermofisher) was incubated into the flow channel for 5min. Excess was removed using 100 μL T50 (50mM Tris-HCl, pH 8.0, 50 mM NaCl). Next, 50 μL of 100pM biotinylated samples was introduced and incubated for 5min in the channel: linear DNA (Fig. 2), triangles (Fig. 3), or biotinylated Anti-His antibody (Fig. S5c and d). Unbound molecules were washed away with 100 μL T50. 100 μL of 10 nM donor labeled imager strands and 100 nM of acceptor labeled imager strands against the sequences under investigation were injected in imaging buffer (50 mM TrisHCl, pH 8.0, 500 mM NaCl, PCD (Merck), PCA (Merck) and 1 mM 6-hydroxy-2,5,7,8-tetramethylchroman-2-carboxylic acid (Trolox) (Sigma)). See Table S1 for the full list of docking and imager strands.

Generally, for single-molecule studies, immobilization is carried out by biotin-streptavidin interactions[4]. However, it is highly difficult to precisely control the number of biotin molecules on the traditionally passivated surfaces (with Biotin-PEG). This raises the possibility of 2 or more binding pockets of streptavidin being occupied by biotins on the slide, leaving only one or two for actual fingerprinting. Thus, we modified the immobilization strategy for tetravalent and divalent streptavidin experiments (Fig. 4): Quartz slides were sonicated for >15min in Acetone, Methanol, and finally 1M KOH with washes with MilliQ in between. Next, the slides were flamed using a burner to remove organic residue if any, and immediately placed back in MilliQ. Finally, the slides were dried using a nitrogen blowgun and used for making the flow cell as explained above. The unused slides were stored at RT. 50μl of 1mg/ml of BSA-Azide (Click chemistry tools, 1535) was incubated with 15μl of 100uM (5') DBCO-DNA-Biotin (3') overnight in the dark at room temperature. 10nM of the resultant BSA-DNA-Biotin was added (50μl total volume) to the flow-cell and incubated for 10min. Excess BSA and free DNA were removed with 100μl T50. Next, 50μl of 1nM tetravalent or divalent Streptavidin was added to the channel and incubated for 5min. The excess was washed with 100μl of T50. Next, 100nM biotinylated docking strands were added to the flow cell and incubated for 30 min to ensure the labeling of all the streptavidin pockets. Unbound DNA was washed away with 100 µL T50. Following, 50 µL of 10 nM donor-labeled imager strands and 100 nM of acceptor-labeled imager strands prepared in the imaging buffer were injected into the flow cell.

Single-molecule fluorescence and FRET data analysis

The data collection and analysis were performed in multiple steps as reported previously[6]. A custom Python script was used to extract time traces of individual molecules from a sCMOS image collected at 0.1s exposure time per frame. Two-state K-means clustering algorithm were applied to the Cy3 and Cy5 fluorescence intensity traces to detect individual binding events of fluorescence imager strands. In order to ensure accurate results, binding events lasting for three or more consecutive frames were selected for further analysis. FRET efficiencies were calculated for each imager strand binding event and used to construct the FRET kymograph and histogram. From the events in which the acceptor probe dissociated or photobleached before the donor probe, we calculated the beta (leakage) and gamma correction factors for accurate FRET efficiency calculation following the method reported in a previous study[7]. Gaussian mixture modeling was applied to automatically classify populations in the FRET histogram. The Python-based automated analysis code can be freely accessed at the following link: https://github.com/kahutia/transient_FRET_analyzer2.

Supplementary Text

Monte Carlo simulations

In *iMAX* FRET, which has multiple identical docking sites, the chance of having single-pair FRET events, i.e. simultaneous binding of one Cy3- and one Cy5 probes, largely depends on the probe binding kinetics. Experimentally, the binding frequency and binding dwell time of a probe can be controlled by the concentration and the length of the DNA probe, respectively. To find the optimal condition that maximizes the chance of having FRET events, we carried out series of Monte Carlo simulations at various kinetic rates. We defined a system with three docking sites each of which had three states of 1) probe unbound, 2) Cy3 probe bound and 3) Cy5 probe bound states. Given transition rates of the two probes, each docking site of the system was allowed to freely transit between states 1 and 2 or states 1 and 3, but not between 2 and 3. Each simulation ran for 1-million-time steps from which we typically observed >5000 transitions. We then selected events in which the system entered into the single-pair FRET emitting state, in which only one Cy3 and Cy5 probe were bound among the three docking sites. After removing events that lasted shorter than three consecutive time steps, the number of the selected single-pair FRET events and the total time spent of the system in them were studied to understand the effect of probe binding kinetics. The simulation code was written in Matlab and freely available upon request.

Structure prediction and classification

A computational pipeline for the reconstruction of 3D-shapes and shape classification was implemented in Python 3.9. Briefly, the number of dyes is determined from the number of FRET efficiency values, which are translated to distances. Distances are used to construct all distinct distance matrices (*D*) using pre-computed index matrices. Each distance matrix is then converted to a coordinate matrix as follows[8]. We construct the Gramm matrix (*M*),

$$M_{ij} = \frac{D_{1j}^2 + D_{i1}^2 - D_{ij}^2}{2}$$

where *i,j* are row and column index respectively. After eigenvalue decomposition,

$$M = USU^T$$

the coordinate matrix *X* can be calculated by sorting U and S by descending order of eigenvalue size, taking the first 3 columns of *U* (*U*[:, :3]) and first 3 eigen values (*S*[:3]) and calculating:

$$X = U[:,:3]\sqrt{S[:3]}$$

Poorly fitting distance matrices generate negative eigenvalues and are excluded. Finally, the remaining coordinate matrices are calculated back to distance matrices, and the coordinate matrix for which distances are closest to the original FRET efficiency-derived distances is returned. The algorithm was implemented in numpy (v1.21.5)[9] with distance matrix calculation as implemented in scipy (v1.8.0)[10].

Numerical embedding of 3D shapes for classification was done using the Geometricus package (v0.3.0)[11]. Embedded coordinates were concatenated to the FRET fingerprint, after which a boosted tree classifier implemented using the XGBoost package (v.1.6.1)[12] was trained and tested on the data using a 10-fold cross validation scheme. The analysis code is freely available at https://github.com/cvdelannoy/iMAX-FRET.

Preparation of the custom DNA nanostructure for Förster radius fitting and classifier applicability analysis

The position of docking site 2 was changed to three different locations (Figure S4a) using click chemistry. To achieve this, alkyne handles were introduced into the DNA backbone at three different locations one at a time in separate constructs. The docking strand for site 2 was designed to contain an azide handle at its one end. The alkyne and azide-containing DNAs were reacted using copper-click chemistry. The clicked DNA products (cyan box, Figure S4b) were gel purified and then the triangles were assembled to generate three structurally similar nanostructures (Figure S4a, bottom left). The positional changes between the (variable) docking site 2 and the fixed docking site 3 were reflected in the FRET values (Figure S4c and d), while it remained constant for all the triangles for the undeviating distance between docking sites three and four (Figure S4e). We could similarly recapitulate the change in FRET values in three coordinates i.e. docking sites two, three and four (Figure S4f).

Conversion of FRET efficiency into the distance R

The following sixth-power relation between *R* and *E* was used to calculate the distance based on the experimentally acquired FRET efficiency.

$$E = \frac{1}{1 + (R/R_0)^6}$$

The Förster radius (R_0), a parameter that combines the influence of dye and medium properties, and relative dye orientations, was fitted using the above custom DNA construct with dyes positioned at known locations along one DNA arm (Figure S4g).

DNA structure modeling for Förster radius fitting

The Förster radius (R_0) denotes the dye distance at which the FRET efficiency is 0.5 and constitutes an essential parameter for the accurate calculation of distances from FRET efficiencies[13]. It factors in dye quantum yields and relative orientations, and the refractive index of the medium. In many applications, it suffices to approximate this value as a constant, however in structural biology, this may lead to unacceptable discrepancies with actual distances, as the effect of local environment and setup is ignored. Here we have used an elegant experiment to determine R_0, using our DNA nanostructure. Briefly, we measure FRET efficiencies for four triangles, created by click-chemistry (detailed in Figure S4).

To determine the Förster radius for our experiments, a single side of the DNA nanostructure was outfitted with clicked docking strands at positions 4, 7, and 15th base from a reference position. FRET efficiencies between clicked docking strands, the reference position, and a third position at one of the other angles of the nanostructure were then measured. We then used a parameter optimization approach with a tree-based Parzen estimator (TPE) implemented in the hyperopt package (v.0.2.7)[14] to estimate the Förster radius. Briefly, this algorithm generates randomized proposals for all one or more variable parameters within given ranges and chooses the combination that minimizes the objective function. The TPE constrains the parameter space based on objective values of previous rounds so that the next guess is more likely to return a lower objective value. Using this approach, we simultaneously fitted Förster radius, linker length, and two DNA geometry parameters (twist and axial rise) after 100 iterations. Here, DNA geometry parameters were allowed to vary slightly to account for unnatural stresses in the nanostructure. As an objective function, the squared sum of the difference between the modeled dye position after triangle construction using given FRET efficiencies (see above) and the expected position given the DNA geometry was used. Table S2 denotes ranges, step sizes, and fitted values for all parameters.

Supplementary Figures

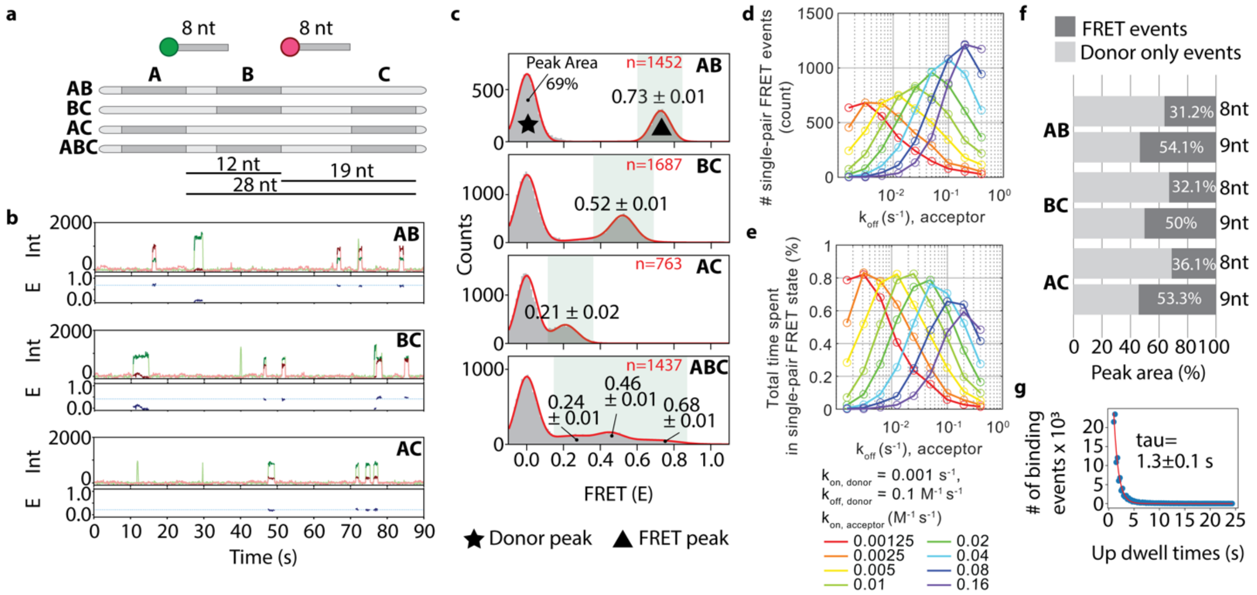

Figure S1: Rational design of the linear construct and imager characteristics for *iMAX* FRET.
a, Schematic representations of the linear DNA constructs. A, B, and C are the positions of identical docking sequences to which 8 nt donor- and acceptor-labeled imagers can bind. The donor to acceptor molar ratio was in a 1:10. The distances between the AB, BC, and AC segments are 12 nt, 19nt, and 28nt, respectively. **b**, Single-molecule intensity time traces for donor (green), acceptor (red) and FRET (blue) for the linear constructs AB, BC, and AC. **c**, Single-FRET event histograms from all molecules in a single field of view. Red solid lines are multi-Gaussian fit to the histograms. The three peaks in the ABC construct correspond to the three distances for A-C (0.24 ± 0.01), B-C (0.46 ± 0.01), and A-B (0.68 ± 0.01) (FRET ± SEM). Star designates the donor-only peak whereas the triangle reports the FRET events peak. **d-e**, The number of single-pair FRET events (d) and the total time spent (e) of a system with three docking strands, obtained from a series of Monte Carlo simulations with various probe binding kinetic rates. Given the donor binding ($k_{on, donor}$ = 0.001) and dissociation ($k_{off, donor}$ = 0.1) rates, the number of single-pair FRET events and total time spent within the state changed substantially with the acceptor binding ($k_{on, acceptor}$) and dissociation ($k_{off, acceptor}$) rates. While the maximum number of events was achieved with higher $k_{on, acceptor}$, the maximum time spent started decreasing when $k_{on, acceptor}$ was more than 10 times higher than that of the donor. At the optimal 10-times higher $k_{on, acceptor}$, $k_{off, acceptor}$ should be ~5-10 times lower than that of the donor to maximize the chance of observing single-pair FRET. **f**, Peak areas for donor only- and single-FRET events for each linear construct are plotted as percentages. Note the increase of 1.5-fold in the FRET events peak area when a longer acceptor imager (9nt) is used instead of an 8nt imager. **g**, Dwell time histogram of the 9nt acceptor imager binding events (Blue circles). The dwell time determined (tau) from single-exponential fit (red line) was 1.3±0.1 s under our experimental conditions (time ± SEM s)

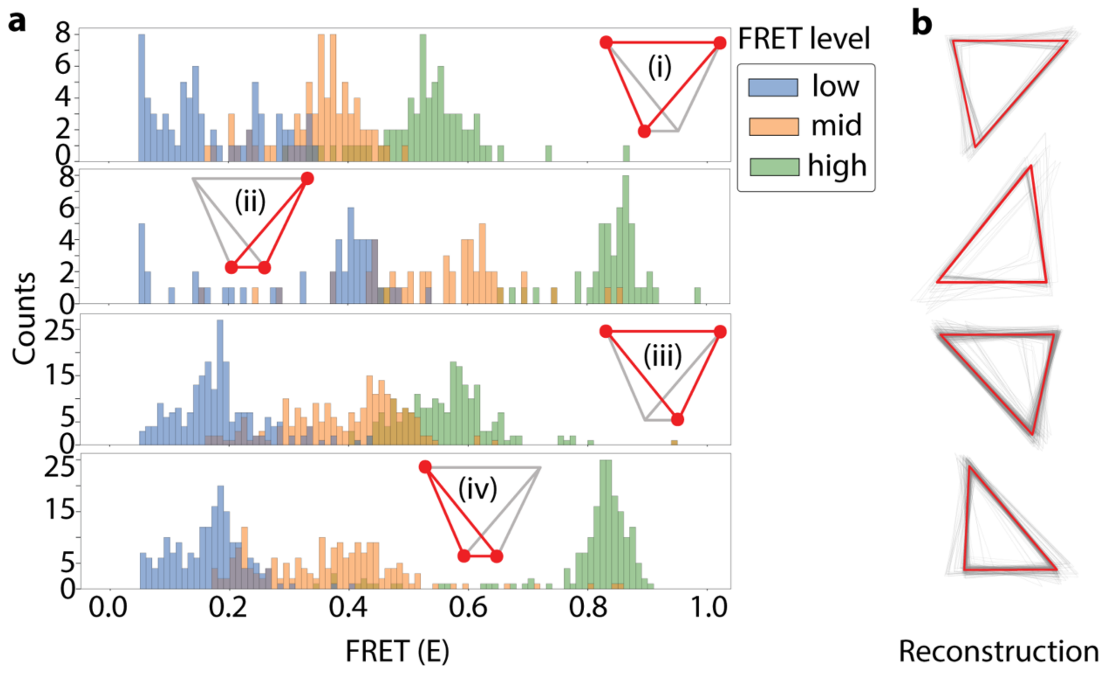

Figure S2: Reconstruction of triangles for single-molecule FRET histograms

a. Histograms displaying per-molecule FRET efficiencies separately for each of the three levels (low, mid, and high) per triangle type in the quadrangular DNA nanostructure (i to iv). Only molecules featuring all three values are shown. **b**. Aligned reconstructed triangles for all single molecules (gray) and the average triangle (red) per triangle type.

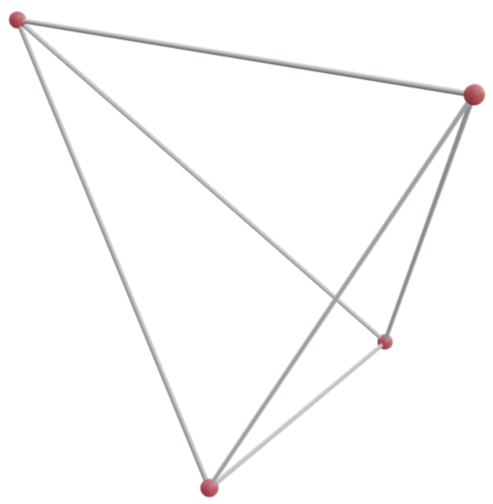

Figure S3: 3D reconstruction of the quadrangular DNA nanostructure
3D reconstruction of the relative dye positions in the nanostructure based on FRET values, revealing its asymmetric and staggered nature.

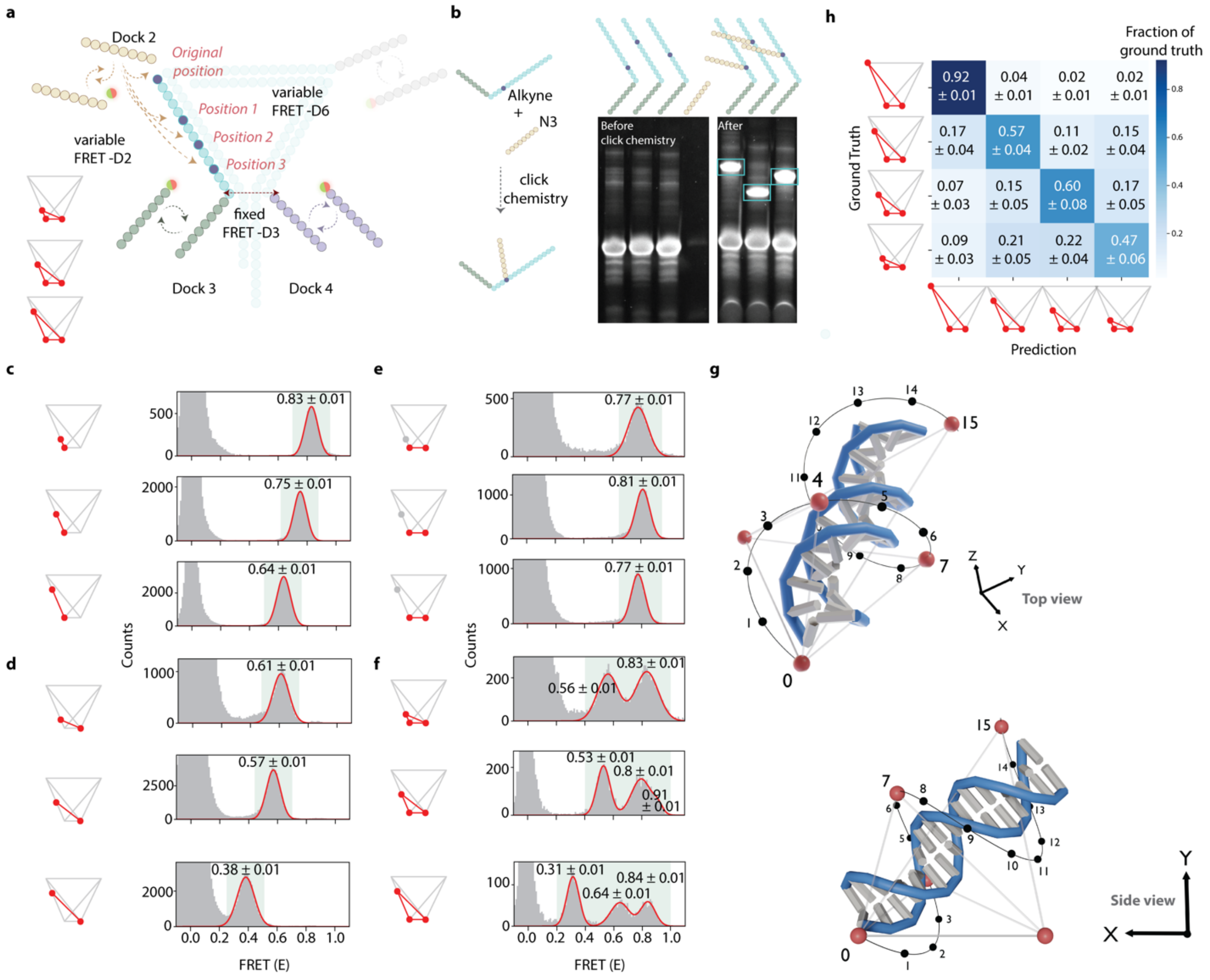

Figure S4: *iMAX* FRET-based analysis of closely related DNA nanostructures

a, In the complex DNA nanostructure, the position of Dock 2 is changed to three different positions giving rise to three FRET-D2 variations. **b**, Click-chemistry is used to attach an azide-linked Dock 3 to the backbone DNA with an alkyne handle. The clicked DNA products (cyan box) were gel extracted and then the nanostructures were reconstituted by hybridization. **c**, The FRET changes between the (variable) Dock 2 and fixed Dock 3 are reflected in the change in the differential position change of Dock 2. **d**, The changes in Dock 2 also changed the distance between sites 2 and 4, as confirmed by the changing FRET values. **e**, FRET values for the distances, between sites 3 and 4, as expected, remained largely unaffected. **f**, The change in FRET values in three points can be similarly recapitulated, for the triangles with imagers and Docks 2,3, and 4. Overall FRET values also shifted for triangles as well for all positions with respect to the original triangle (iv). **g**, 3D reconstruction of dsDNA strand (blue/white) with dye positions (red spheres) of three triangles with the same base reconstructed from FRET efficiencies. Triangles differed in the position of their third dye, which was located at nucleotides with indices 4, 7, or 15, counted from the base. Förster radius, DNA twist, DNA axial rise, and dye-DNA linker length were optimized using a tree-based Parzen estimator-based approach. Black numbers and dots denote expected dye positions and indices for linkers attached to different nucleotides, based on DNA geometry and linker length. Images rendered at two different view angles were generated in Blender (v3.6). **h**, our integrated computational approach can differentiate the 3D structures from each other on a single molecule level with up to 60% accuracy.

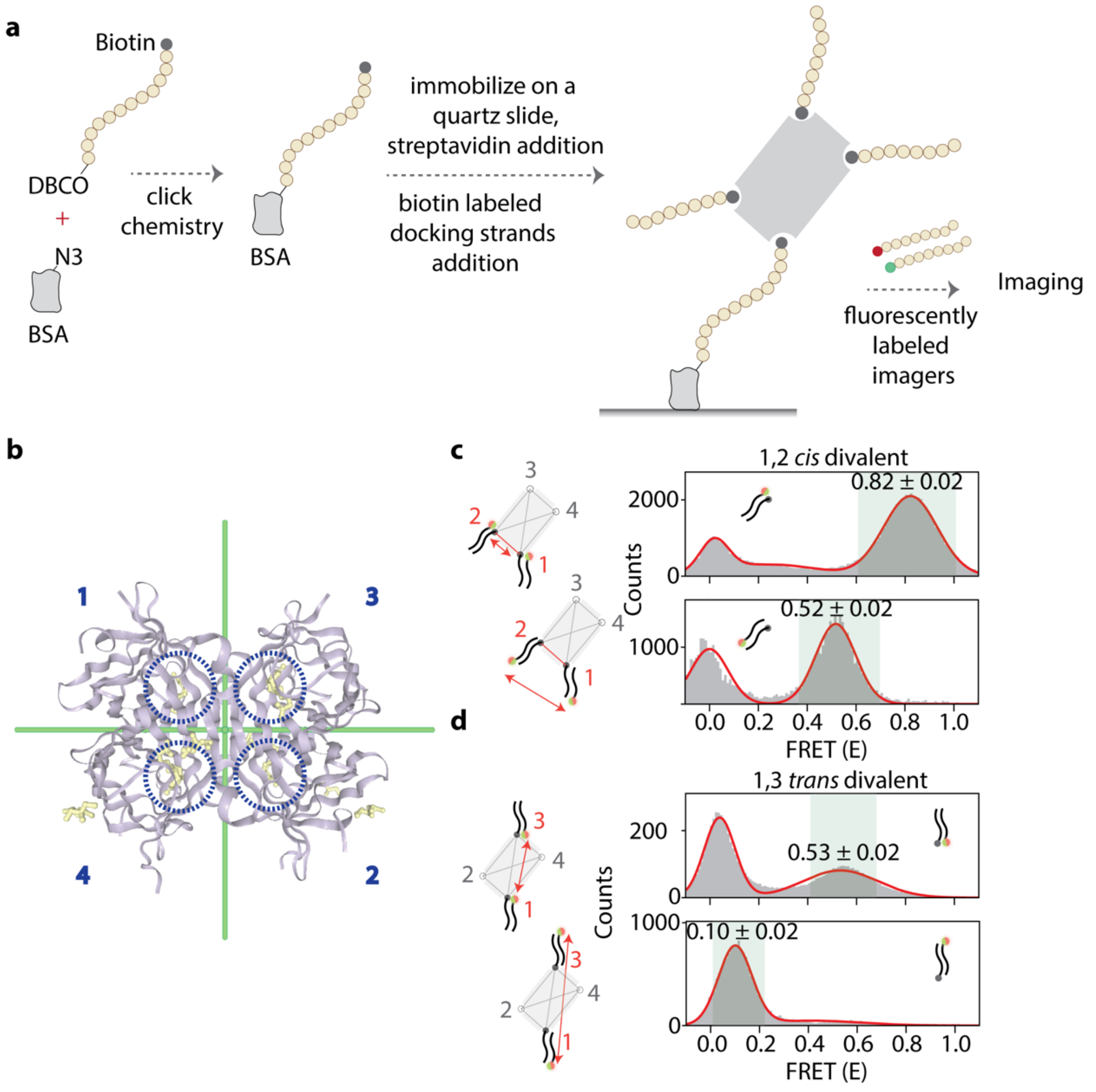

Figure S5: Immobilization scheme of streptavidins and their structural analysis

a, BSA-Azide was immobilized on a quartz slide, conjugated with DNA with a DBCO handle at one end and biotin at the other. The presence of only one Azide per BSA molecule allowed the attachment of one biotin, and thus one streptavidin molecule per BSA molecule. Using this newly developed immobilization scheme, we could ensure that only one pocket is filled with biotin for immobilization and that the remaining 3 pockets are available for binding biotinylated docking sequences for fingerprinting. **b**, D2 symmetry of the wild-type streptavidin tetramer (from PDB ID: 3RY2). 1,2,3 and 4 designate the numbering of subunits in the tetramer. Biotins (yellow space fills) are highlighted with dashed blue circles. **c and d**, With the use of imagers for probing as opposed to covalently conjugated dyes generally used in FRET assays, we could modify the location of dye to artificially change the distance between the 2 points. When 3' instead of 5' dye-labeled imagers were applied, we could see the relative FRET shift to lower efficiencies corresponding to the new distance, on the divalent structures. 1,2 cis divalent streptavidin shows a change of 0.30 FRET value, while it is 0.43 for the 1,3 trans divalent streptavidin mutants.

Supplementary Tables

Table S1: DNA constructs

Corresponding Figure	Description	Sequence (5'-3')	Modification	Supplier
Figure 2	Linear construct with POI-A and B docking sequences	TTTTTTTTTTTTTTTTTTTTATAC ATCTATTTATACATCTA	5' Biotin	Ella Biotech (GmbH)
Figure 2	Linear construct with POI-B and C docking sequences	TTTTTATACATCTATTTTTTTA TACATCTATTTTTTTTTT	5' Biotin	Ella Biotech (GmbH)
Figure 2	Linear construct with POI-A and C docking sequences	TTTTTATACATCTATTTTTTTTT TTTTTTTTATACATCTA	5' Biotin	Ella Biotech (GmbH)
Figure 2	Linear construct with POI-A, B and C docking sequences	TTTTTATACATCTATTTTTTTA TACATCTATTTATACATCTA	5' Biotin	Ella Biotech (GmbH)
Figure 2	Donor imager strand	AGATGTAT	3' Cy3	Ella Biotech (GmbH)
Figure 2	Acceptor imager strand	AGATGTAT	3' Cy5	Ella Biotech (GmbH)
Figure 2	Longer acceptor imager strand	TAGATGTAT	3' Cy5	Ella Biotech (GmbH)
Figure 3	DNA Nanostructure backbone + Dock 1	ATTCATTCTCATCCTCTGTCG GGTGTACCGTAAGGTGAAT AGTGACTTTATACATCTA	-	Ella Biotech (GmbH)
Figure 3	DNA Nanostructure Left arm + Dock 2 and Dock 3	AGAGGAGGATTTCGGTACAC CCGACAGTTTTCAATGTA	-	Ella Biotech (GmbH)
Figure 3	DNA Nanostructure right arm + Dock 4	TCTTCATTACTTTTCGATAA CAATCAGGTCACTATTCACC TTA	-	Ella Biotech (GmbH)
Figure 3	DNA Nanostructure biotin strand	CTGATTGTTATCGAGGATGA GAATGAATTTTTTTTTTTTTT TTT	Biotin – 3'end labeled	Ella Biotech (GmbH)
Figure 3	DNA Nanostructure donor imager strand Dock 1	AGATGTAT	3' Cy3	Ella Biotech (GmbH)
Figure 3	DNA Nanostructure acceptor imager strand Dock 1	TAGATGTAT	3' Cy5	Ella Biotech (GmbH)
Figure 3	DNA Nanostructure donor imager strand Dock 2	TCCTCCT	5' Cy3	Ella Biotech (GmbH)

Figure 3	DNA Nanostructure acceptor imager strand Dock 2	TCCTCCTC	5’ Cy5	Ella Biotech (GmbH)
Figure 3	DNA Nanostructure donor imager strand Dock 3	TACATTGA	3’ Cy3	Ella Biotech (GmbH)
Figure 3	DNA Nanostructure donor imager strand Dock 3	TACATTGAA	3’ Cy5	Ella Biotech (GmbH)
Figure 3	DNA Nanostructure donor imager strand Dock 4	AGTAATGA	5’ Cy3	Ella Biotech (GmbH)
Figure 3	DNA Nanostructure acceptor imager strand Dock 4	AGTAATGAAG	5’ Cy5	Ella Biotech (GmbH)
Figure S4	Clickable DNA Nanostructure Left arm + Dock 2 Position 2	CGGTA7ACCCGACAGTTTTCA ATGTA	7= C8-Alkyne-dC	Biomers.net (GmbH)
Figure S4	Clickable DNA Nanostructure Left arm + Dock 2 Position 3	CGGTACACC7GACAGTTTTCA ATGTA	7= C8-Alkyne-dC	Biomers.net (GmbH)
Figure S4	Clickable DNA Nanostructure Left arm + Dock 2 Position 4	CGGTACACCCGA7AGTTTTCA ATGTA	7= C8-Alkyne-dC	Biomers.net (GmbH)
Figure S4	Clickable Dock 2	AGAGGAGGATTT	5’ Azide-pro	Biomers.net (GmbH)
Figure 4, Figure S5	Docking strand for streptavidin WT and mutants	ATACATCTA	3’ Biotin	Ella Biotech (GmbH)
Figure 4, Figure S5	Immobilization strand for streptavidin WT and mutants	AAAAGAAAAGAAATACATCT AT	5’ DBCO, 3’ Biotin	Ella Biotech (GmbH)
Figure 5	Docking strand for proteins – SBD2 WT and mutants	TATACATCTAT	5’ Azide-pro	Ella Biotech (GmbH)

Table S2: **Förster radius fitting parameters**

Ranges, step sizes, and fitted values for all parameters fitted by the tree-based Parzen estimator optimization algorithm. Here, the structure diameter spans the DNA strand diameter and two times the linker length.

	Min	Max	Step size	Fitted value
Förster radius (Å)	50	60	0.1	53.8
DNA twist (°/bp)	32	40	1	39
Axial rise (Å/bp)	2.3	5.0	0.1	4.1
Structure diameter (Å)	25	70	0.5	38

References

(1) Fairhead, M.; Krndija, D.; Lowe, E. D.; Howarth, M. Plug-and-play pairing via defined divalent streptavidins. *J Mol Biol* **2014**, *426* (1), 199-214. DOI: 10.1016/j.jmb.2013.09.016.
(2) de Boer, M.; Gouridis, G.; Vietrov, R.; Begg, S. L.; Schuurman-Wolters, G. K.; Husada, F.; Eleftheriadis, N.; Poolman, B.; McDevitt, C. A.; Cordes, T. Conformational and dynamic plasticity in substrate-binding proteins underlies selective transport in ABC importers. *eLife* **2019**, *8*. DOI: 10.7554/eLife.44652.
(3) Kantner, T.; Alkhawaja, B.; Watts, A. G. In Situ Quenching of Trialkylphosphine Reducing Agents Using Water-Soluble PEG-Azides Improves Maleimide Conjugation to Proteins. *ACS Omega* **2017**, *2* (9), 5785-5791. DOI: 10.1021/acsomega.7b01094.
(4) Roy, R.; Hohng, S.; Ha, T. A practical guide to single-molecule FRET. *Nat Methods* **2008**, *5* (6), 507-516. DOI: 10.1038/nmeth.1208.
(5) Kapanidis, A. N.; Laurence, T. A.; Lee, N. K.; Margeat, E.; Kong, X.; Weiss, S. Alternating-laser excitation of single molecules. *Acc Chem Res* **2005**, *38* (7), 523-533. DOI: 10.1021/ar0401348.
(6) Filius, M.; Kim, S. H.; Severins, I.; Joo, C. High-Resolution Single-Molecule FRET via DNA eXchange (FRET X). *Nano Lett* **2021**, *21* (7), 3295-3301. DOI: 10.1021/acs.nanolett.1c00725.
(7) McCann, J. J.; Choi, U. B.; Zheng, L.; Weninger, K.; Bowen, M. E. Optimizing methods to recover absolute FRET efficiency from immobilized single molecules. *Biophys J* **2010**, *99* (3), 961-970. DOI: 10.1016/j.bpj.2010.04.063.
(8) Crippen, G. M., Havel, T. F., . Stable calculation of coordinates from distance information. *Acta Crystallographica Section A* **1978**, *2* (34), 282-284. DOI: 10.1107/S0567739478000522.
(9) Harris, C. R.; Millman, K. J.; van der Walt, S. J.; Gommers, R.; Virtanen, P.; Cournapeau, D.; Wieser, E.; Taylor, J.; Berg, S.; Smith, N. J.; et al. Array programming with NumPy. *Nature* **2020**, *585* (7825), 357-362. DOI: 10.1038/s41586-020-2649-2.
(10) Virtanen, P.; Gommers, R.; Oliphant, T. E.; Haberland, M.; Reddy, T.; Cournapeau, D.; Burovski, E.; Peterson, P.; Weckesser, W.; Bright, J.; et al. SciPy 1.0: fundamental algorithms for scientific computing in Python. *Nat Methods* **2020**, *17* (3), 261-272. DOI: 10.1038/s41592-019-0686-2.
(11) Durairaj, J.; Akdel, M.; de Ridder, D.; van Dijk, A. D. J. Geometricus represents protein structures as shape-mers derived from moment invariants. *Bioinformatics* **2020**, *36* (Suppl_2), i718-i725. DOI: 10.1093/bioinformatics/btaa839.
(12) Guestrin, T. C. a. C. XGBoost: A Scalable Tree Boosting System. *arXiv* **2016**. DOI: 10.1145/2939672.2939785. (accessed 2024.01.26).
(13) Hohlbein, J.; Craggs, T. D.; Cordes, T. Alternating-laser excitation: single-molecule FRET and beyond. *Chem Soc Rev* **2014**, *43* (4), 1156-1171. DOI: 10.1039/c3cs60233h.
(14) Bergstra, J.; Yamins, D.; Cox, D. Making a Science of Model Search: Hyperparameter Optimization in Hundreds of Dimensions for Vision Architectures. In Proceedings of the 30th International Conference on Machine Learning, Proceedings of Machine Learning Research; 2013.
